# Effect of Zolpidem in the Aftermath of Traumatic Brain Injury: An MEG Study

**DOI:** 10.1155/2020/8597062

**Published:** 2020-03-20

**Authors:** Praveen Sripad, Jessica Rosenberg, Frank Boers, Christian P. Filss, Norbert Galldiks, Karl-Josef Langen, Ralf Clauss, N. Jon Shah, Jürgen Dammers

**Affiliations:** ^1^Institute of Neuroscience and Medicine (INM-4), Medical Imaging Physics, Forschungszentrum Jülich GmbH, 52425 Jülich, Germany; ^2^Institute of Neuroscience and Medicine (INM-11), JARA, Forschungszentrum Jülich GmbH, 52425 Jülich, Germany; ^3^Department of Neurology, RWTH Aachen University, Aachen, Germany; ^4^Department of Nuclear Medicine, RWTH Aachen University, Aachen, Germany; ^5^Institute of Neuroscience and Medicine (INM-3), Forschungszentrum Jülich, Jülich GmbH, 52425 Jülich, Germany; ^6^Department of Neurology, University of Cologne, Cologne, Germany; ^7^Center of Integrated Oncology (CIO), Universities of Cologne and Bonn, Cologne, Germany; ^8^Nuclear Medicine Department, Royal Surrey County Hospital, Guildford, Surrey GU2 7XX, UK; ^9^JARA - BRAIN - Translational Medicine, Aachen, Germany

## Abstract

In the past two decades, many studies have shown the paradoxical efficacy of zolpidem, a hypnotic used to induce sleep, in transiently alleviating various disorders of consciousness such as traumatic brain injury (TBI), dystonia, and Parkinson's disease. The mechanism of action of this effect of zolpidem is of great research interest. In this case study, we use magnetoencephalography (MEG) to investigate a fully conscious, ex-coma patient who suffered from neurological difficulties for a few years due to traumatic brain injury. For a few years after injury, the patient was under medication with zolpidem that drastically improved his symptoms. MEG recordings taken before and after zolpidem showed a reduction in power in the theta-alpha (4–12 Hz) and lower beta (15–20 Hz) frequency bands. An increase in power after zolpidem intake was found in the higher beta/lower gamma (20–43 Hz) frequency band. Source level functional connectivity measured using weighted-phase lag index showed changes after zolpidem intake. Stronger connectivity between left frontal and temporal brain regions was observed. We report that zolpidem induces a change in MEG resting power and functional connectivity in the patient. MEG is an informative and sensitive tool to detect changes in brain activity for TBI.

## 1. Introduction

Zolpidem is a short-acting nonbenzodiazepine hypnotic that is commonly used to treat insomnia [1] and has been shown to be highly selective for the GABA-A receptor [2]. To the best of our knowledge, Daniele and colleagues were the first to report a seemingly paradoxical therapeutic effect on a patient with Parkinson's disease [3]. They reported a 61-year-old woman with a 25-year history of Parkinson's who showed antiparkinsonian improvements in akinesia and rigidity, instead of drowsiness upon a 10 mg dosage of zolpidem. Findings stemming from a case study of a patient in the persistent vegetative state followed, where a patient woke up from his semi-coma and could recognize and talk to his mother after the intake of zolpidem [4]. Since then, there have been many reports where zolpidem has been used to treat a variety of neurological disorders of consciousness [5–10]. In a systematic review, Bomalaski and colleagues discussed the literature regarding the potential of zolpidem to treat a variety of neurological disorders and concluded that more research is needed to interrogate the complex underlying neurophysiological mechanisms involved and in order to understand zolpidem's usefulness as a therapeutic drug [11].

It is well known that resting state magnetoencephalography (MEG) is a useful tool in understanding function and dysfunction in the human brain [12]. Slow and fast oscillatory changes in the resting state functional connectivity during MEG have been shown to reflect various brain disorders such as stroke, epilepsy [13], traumatic brain injury (TBI) [14], Alzheimer's disease [15], and autism spectrum disorders [16]. Thus, looking at large-scale MEG resting state networks may give us an insight into the functional organisation of the injured brain and the effects of various drugs on it. It has been shown that MEG can pick up changes in GABA function in the brain and that zolpidem has an influence on changes in GABA action [17]. This makes the analysis of resting state brain oscillations using MEG particularly relevant to understanding the effects of zolpidem.

Unfortunately, there are only a limited number of studies using MEG or electroencephalogram (EEG) to observe changes in brain activity following treatment with zolpidem to relieve neurological symptoms. However, one study conducted by Hall and colleagues used MEG (along with fMRI, MRS, and SPECT) (functional magnetic resonance imaging, magnetic resonance spectroscopy, and single-photon emission computed tomography) to observe coincidental sensorimotor and cognitive improvements in a stroke patient [18]. The authors reported reduced MEG signal power in the theta (4–10 Hz) and beta (15–30 Hz) bands after zolpidem uptake. Subsequently, Williams and colleagues investigated the resting state brain using EEG in order to identify a possible mechanism underlying zolpidem response after brain injury and reported reduced EEG power and coherence at low frequencies (6–10 Hz) [19]. A number of other studies using EEG reported incomplete or inconclusive results [20–22]. In a structured study, Whyte and Myers investigated 84 participants with disorders of consciousness and reported that 4.8% of the participants were responsive to zolpidem [23]. The relative rarity of such cases and a lack of understanding of the mechanisms involved are a strong motivation for continued research.

Here we report differences in the spectral power and MEG functional connectivity at the source level using weighted phase lag index (WPLI) [24] in the resting state brain recordings of the patient before and after intake of zolpidem.

## 2. Case Presentation

**Figure 1:**
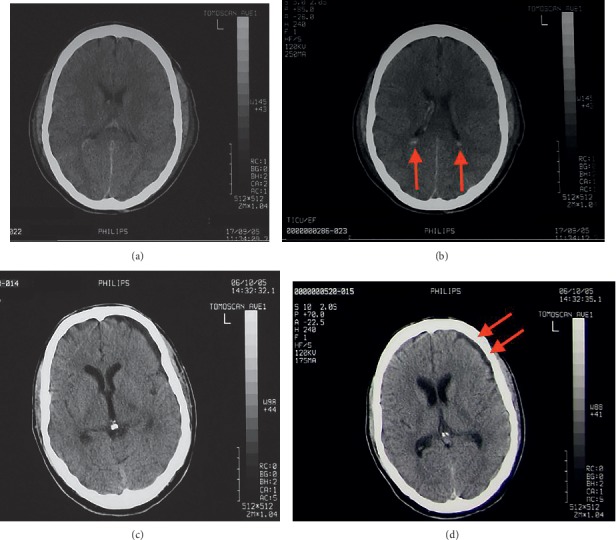
CT scans of the patient brain immediately after (a and b) and 19 days after accident (c and d). The images (a) and (b) above show some intraventricular hemorrhage (arrows show intraventricular hemorrhage in the posterior horns of the lateral ventricles) after accident. In the images (c) and (d), the intraventricular hemorrhage has resolved completely. Arrows show suspected new discrete atrophic changes in the left frontal lobe with sulcal prominence that was not present in the initial scans.

The subject (male, 35), henceforth referred to as W, suffered from a severe traumatic brain injury to the left side of his brain in a major car accident in 2005. Based on computerized tomography (CT) scans of the brain, W suffered from intraventricular hemorrhage that was resolved within 19 days of the accident. A very small left fronto-temporal collection not causing obvious mass effect was suspected along with some atrophic change in the inferior aspect of the left frontal lobe with sulcal prominence inferiorly.  Figure 1 shows the CT scans of the patient brain immediately after the accident and 19 days later. After the accident, he remained in a coma for a few weeks, progressing to a vegetative state, and eventually gained full consciousness three months after the accident. After a phase of hospital rehabilitation, he returned home after a year. The accident left him disabled with difficulty walking and a limp. His coordination, short-term memory, and speech (particularly pronunciation of consonants) were also affected, leading to lack of confidence in performing daily activities. In 2011, it was discovered that these deficits markedly improved following the administration of a 10 mg dose of zolpidem. At this stage, a neurological examination showed that W was right-handed, normally oriented in time, place, and person. W had a severe dysarthria and an impaired uvula elevation, but no dysphagia. The left biceps and brachoradialis reflexes were absent, and the left triceps reflex impaired. There was a bilateral brady-dysdiadochokinesia, more pronounced left, and dysmetria in the finger-to-nose and heel-to-shin tests. There was a broad-based gait, impaired tandem walking, an abnormal postural response, and a broad-based stance. The Romberg test was abnormal. The unified Parkinson's disease rating scale (UPDRS) measured 10, and the Tinetti and Barthel scores were 21 and 10, respectively [25–27]. After intake of zolpidem, W's speech, movement, coordination, and gait showed drastic improvements. The improvements started approximately 30 minutes after ingestion and lasted up to 4 hours, with a maximum effect after around 1 hour. While W's Barthel index remained normal before and after zolpidem, his ability on the Tinetti Falls Efficacy Scale improved from 21/100 to 15/100. A baseline ^99m^Tc-HMPAO brain SPECT scan in 2011 showed a decreased cerebral perfusion in the left fronto-parietal and temporal regions and the left thalamus, which improved after zolpidem intake. Upon starting 10 mg of zolpidem daily, W's speech, movement, coordination, and gait improved, leading to him being able to lead a near normal life. After two years, along with the MEG measurements, ^18^F-FDG and ^11^C-flumazenil PET scans were performed before and after zolpidem intake and showed no abnormalities in W's brain. A 99m Tc-HMPAO brain SPECT scan repeated in 2014 appeared normal and showed no changes upon zolpidem intake. At the time of the MEG measurement (used in this study) in November 2013, W was under 10 mg of zolpidem daily. The MEG measurements were obtained during a no-task resting condition with eyes open (EO) and eyes closed (EC) for three minutes. The first measurement was taken without W having taken zolpidem for 24 hours. Subsequently, 10 mg of zolpidem was administered and measurements were taken an hour later. Thus W was measured under two conditions: without any zolpidem (NOZO) and with zolpidem (ZO). Voluntary consent from the subject was obtained before performing the measurement, and the measurement was conducted in the presence of the subject's supervising physician.

## 3. Results

Previously in 2011, following intake of zolpidem, W had shown marked improvements in speech, movement, gait, and coordination. However, at the time of measurement in 2013, upon neurological examination, the patient showed no clinical effects following intake of zolpidem and only minor subjective improvements were reported. For example, W's family felt that he functioned better when on zolpidem. A structural MR scan performed at the time of MEG measurement showed no major structural abnormalities.

**Figure 2:**
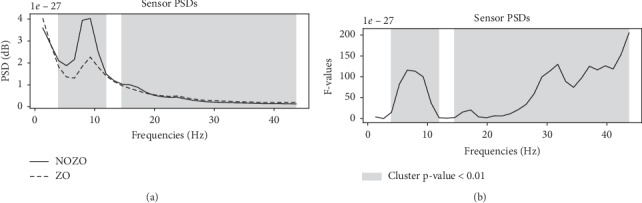
Significant differences in the sensor level PSDs in the subject with (ZO) and without (NOZO) zolpidem drug. Panel (a) shows average PSDs across trials with (dashed line) and without zolpidem (solid line). Panel (b) shows the F-statistic across frequencies. Frequency clusters for which the PSD differences are statistically significant across trials with a *p* value of 0.01 as shown in gray.

**Figure 3:**
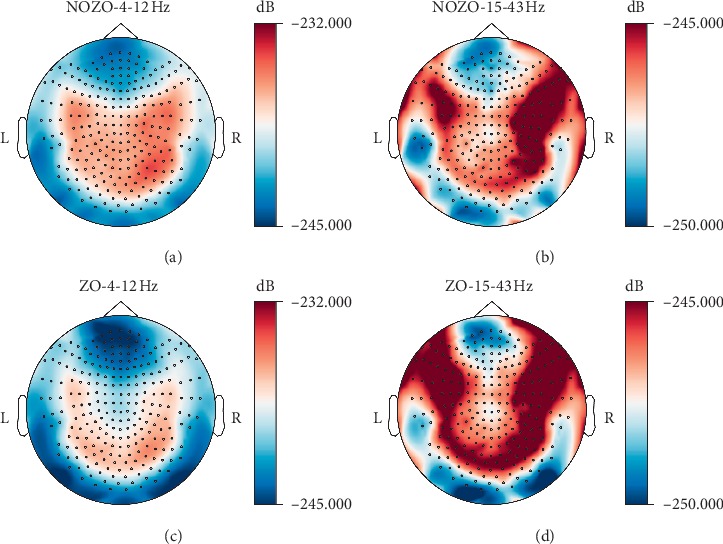
Power spectral densities across the sensor topography for 4–12 Hz and 15–43 Hz frequency bands for the patient with (ZO) and without Zolpidem (NOZO). Differences in PSD values in the left frontal areas (neurological orientation) before and after ZO under frequency 15–43 Hz can be seen.

MEG power spectral densities at the sensor level analysis were computed to identify differences, if any, in the subject before and after zolpidem intake. As shown in Figure 2, a permutation cluster test [28] showed a significant decrease (*p* < 0.01) in power in the frequency range of 4–12 Hz and 15–20 Hz, whereas an increase can be observed above 20 Hz up to 43 Hz (see Figure 3).

Sensor level topomaps computed across the frequency bands of interest before and after intake of ZO shows how the power is distributed across the sensors. A clear decrease in power is observed after ZO for the frequency band 4–12 Hz. The 15–43 Hz frequency bands show an increase in power, especially in the sensors above the left frontal areas of the brain.

**Figure 4:**
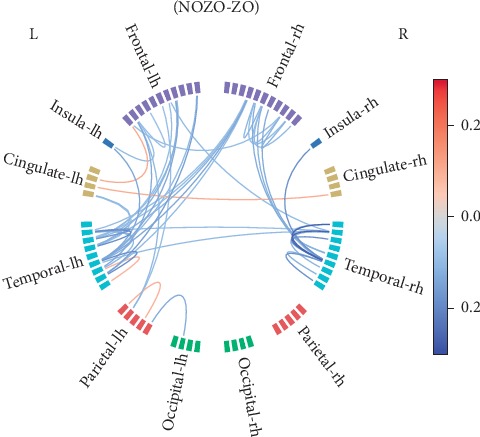
Connectivity difference before (NOZO) and after the intake of zolpidem (ZO) in the 15–43 Hz frequency band. The 50 strongest connections are visualized, where reddish lines indicate stronger connections without zolpidem and bluish lines indicate stronger connections with zolpidem.

Connectivity analysis was performed on representative time courses from different brain regions [29] obtained from MEG data projected onto the source space using minimum norm estimates (MNE) [30]. WPLI was used to quantify the connectivity strength between brain regions. In Figure 4 a circle plot shows the contrast of WPLI strength between NOZO and ZO conditions for different brain areas in the 15–43 Hz frequency band. The areas, corresponding to the 68 anatomical labels, are grouped according to cortical regions and color-coded accordingly. The strength of connectivity (WPLI) is indicated using the color of the lines connecting various areas. Differences in connections between the frontal and temporal regions that are absent in NOZO show up strongly (blue lines) in the ZO condition, particularly in the left hemisphere.

## 4. Discussion

W, suffering from several neurological disabilities as an aftermath of TBI showed drastic improvement in speech and motor function after the intake of zolpidem in low doses. This transient counterintuitive effect of zolpidem, a commonly used hypnotic, has been observed in many people with a variety of neurological disorders. While this effect has been well documented in the literature, much still remains to be understood [11].

In this case study, we have measured the resting state brain activity in W using ME and have documented the differences in source power and functional connectivity before and after intake of zolpidem.

Strong differences in power were found at the sensor level. A spectral permutation clustering between trials showed a significant reduction in power after intake of zolpidem between 4 and 12 Hz, and from 15 to 20 Hz that includes the theta, alpha, and lower beta range. We find that this is in agreement with previous studies using MEG and EEG [18, 19]. In a case report describing the paradoxical effects of zolpidem in a patient in a persistent vegetative state, Hall and colleagues demonstrated high theta (4–10 Hz) and beta (15–30 Hz) MEG power that decreased upon zolpidem uptake. This was also coupled with improvements in cognitive and motor function in the patient [18]. Williams and colleagues reported a similar reduction in 6–10 Hz EEG power in several subjects with brain injury [19]. The effect has also been observed in healthy subjects in both M/EEG [2, 17] and in functional magnetic resonance imaging (fMRI) [31]. It is particularly relevant that among other regions, Licata and colleagues also report a strong increase in the resting state blood oxygen level-dependent (BOLD) response after zolpidem intake in the occipital area [31]. The anticorrelation between MEG power and BOLD, especially in the occipital cortex is well documented [32, 33]. Our results also show an increase in power in the lower gamma range that has not been reported thus far. The topomaps show asymmetrically stronger power in the right hemisphere before zolpidem intake that disappears after zolpidem. This may be explained by the fact that W suffered from a left-sided head TBI, with some dormancy of function observed in the left fronto-parietal, temporal, and thalamic regions (as reported by the ^99m^Tc-HMPAO brain SPECT scans from his physician).

The functional connectivity analysis results show variation in connectivity after zolpidem intake with the frontal and temporal regions of the left hemisphere being most prominent. Studies on healthy subjects using MEG show that frontal and temporal areas are actively involved in the resting state [34]. Furthermore, zolpidem is a well-known positive modulator of *γ*-aminobutyric acid_A_ (GABA_A_) receptors [1, 35], and studies on healthy populations have shown that GABA levels are correlated to functional connectivity [36]. Zolpidem increased functional connectivity in the resting state networks of healthy participants measured using fMRI BOLD time-series [31]. A number of hypotheses have been proposed to understand the mechanism of the action of zolpidem and to explain the functional significance of its effects in clinical observations [37–39].

It is important to note that, at the time of measurement, the patient was on a daily dose of zolpidem for two years and was zolpidem free for a period of 24 hours before start of the experiment, raising the possibility that the results are confounded due to tolerance effects. The half-life of zolpidem is around 2.4 hours. A 24 hours zolpidem free period translates to ten half lives which is enough time for the effects of the previous dose to wash out of the system. As far as the long-term tolerance effects of zolpidem are concerned, the current body of literature is not very clear. A meta-analysis from 1999 has shown that zolpidem produces only a minimum tolerance in the long run [40]; however, the analysis did not include a contradictory study where tolerance to the hypnotic effects of zolpidem was reported [41]. A later study from 2012 reports no tolerance on insomnia to zolpidem after a year of use [42]. When W was first introduced to zolpidem medication in 2011, a ^99m^Tc-HMPAO brain SPECT scan showed decreased cerebral perfusion with transient improvement after zolpidem. A subsequent scan in 2014 showed a normal perfusion with no significant change after zolpidem. Positron emission tomography (PET) measurements with ^18^F-FDG and ^11^C-Flumazenil conducted in 2013 around the same time as the MEG measurements showed normal glucose metabolism and benzodiazepine receptor distribution without significant changes after zolpidem. These unpublished results from the SPECT and PET scans suggest that W may have shown secondary long-term improvement parallel to that of daily transient improvements induced by zolpidem. Considering that the literature is unclear in terms of tolerance caused by zolpidem, we hypothesize that the patient has undergone a partial healing over the years rather than develop a tolerance to the drug. Therefore, there is a possibility that a part of the changes observed would be expected to occur in the healthy brain as well. This gradual process of natural recovery has also been observed in other patients with TBI too [43].

Furthermore, it has to be considered that the changes in spectral power and functional connectivity analysis of MEG data after administration of zolpidem could not be substantiated by the neurological examination. Previous studies have shown that MEG is a very sensitive method to detect functional changes in the brain of TBI patients as compared to EEG, SPECT, and MRI [44–46]. In their 1999 study, Lewine and colleagues have shown MEG abnormalities even in an asymptomatic patient with a history of mild TBI as compared to controls. Similarly, Tarapore and colleagues show disrupted MEG functional connectivity in patients years after TBI, even in patients with normal MRI findings [46]. Based on these observations, it is reasonable to assume that the functional changes observed with MEG in our study are real, even though they are not directly substantiated by the neurological examination. In this context, we consider the subjective improvements reported by the patient and his family as confirmation, but not as proof of the functional changes.

From the above findings we may state that zolpidem produces a transient change in the MEG resting state spectral power and functional connectivity in the patient. We hypothesize that although damage to the brain has slowly healed over the time; there may remain decreased cortical network activity that is improved upon zolpidem intake. The extent of zolpidem-induced changes observed may be a function of time since injury, and therefore it is important to keep this in mind when interpreting the results and in comparing different studies. Abnormally reduced connectivity in TBI, observed via resting state MEG has been shown to improve over time [46]. Longitudinal studies involving the effect of zolpidem on an injured brain would also help further understanding whether daily doses of zolpidem would have any long-term influence in aiding recovery. Studies with a larger cohort looking at the influence of zolpidem on healthy subjects using resting state MEG would also greatly help understand the mechanisms involved.

## 5. Conclusion

We have reported findings from a case of traumatic brain injury where zolpidem produced transient paradoxical improvements in a patient with neurological disabilities. We report a reduction in MEG power in the theta-alpha band (4–12 Hz) and an increase in the frequency band (20–43 Hz) after zolpidem intake. Changes in the cortico-cortical functional connectivity after zolpidem intake were also observed. Our findings support the assumption that zolpidem has an effect on the neuronal activity. However, this needs to be evaluated further in a larger cohort in order to be helpful for the future treatment of patients affected by such neurological disorders. We also highlight the efficacy of resting state MEG as an investigative tool for TBI.

## Supplementary Materials

Supplementary Materials The supplementary materials contain the results obtained from other imaging modalities (CT, SPECT, PET, and MRI) conducted on the patient. A short description of the imaging methods used is also included. The images show differences in the brain scans of the patient across time with and without Zolpidem.
