# Supplementary material for: Effect of Zolpidem in the Aftermath of Traumatic Brain Injury: An MEG Study
Source: Case Rep Neurol Med. 2020 Mar 20;2020:8597062. doi: 10.1155/2020/8597062 (PMC7109561; doi:10.1155/2020/8597062)
Supplement: Supplementary Materials — The supplementary materials contain the results obtained from other imaging modalities (CT, SPECT, PET, and MRI) conducted on the patient. A short description of the imaging methods used is also included. The images show differences in the brain scans of the patient across time with and without Zolpidem. [file 8597062.f1.docx]

**Supplementary materials**

**Imaging results**


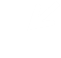

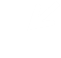

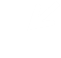

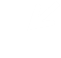

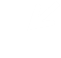

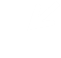

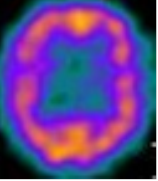

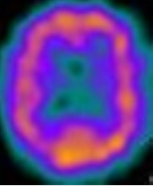

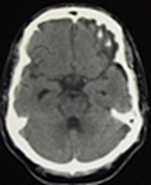

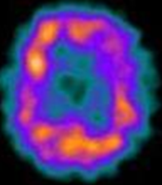

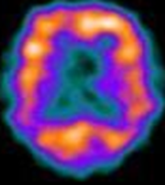


2005

CT scan

NOZO

ZO

2011

^99m^Tc HMPAO SPECT scan

2013

^18^F

FDG

PET scan

2013

^11^C Flumazenil

PET scan

2013

MRI scan

2014

^99m^Tc HMPAO SPECT scan

Figure 1: Serial imaging from 2005 to 2014, showing a transaxial slice of W’s brain with a lesion located left frontally (radiological orientation). The top row shows the scans off zolpidem (NOZO), the lower on zolpidem (ZO).


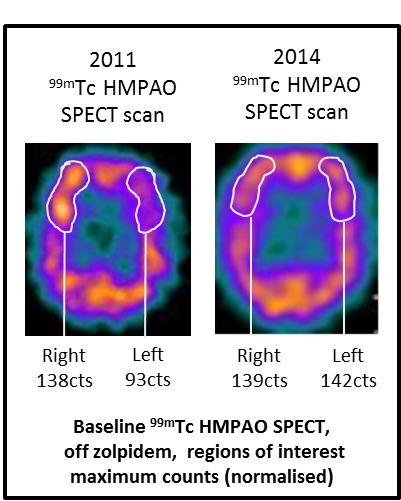


Figure 2: A transaxial slice through W’s brain injury area shows a permanently improved baseline cerebral perfusion in 2014, compared to 2011. The slice shows the activity (maximum counts) in the left sided brain injury region versus the contralateral uninjured side.

**Imaging methods**

The baseline ^99m^Tc-HMPAO brain SPECT scan was performed with an intravenous injection of 740 MBq ^99m^Tc-HMPAO one hour before the scan on a Siemens ECAM SPECT system, set at 120 views, 20 seconds each.

The ^18^F-FDG-PET/MR brain scans commenced at a peripheral blood glucose level of <150 mg/dL before injection of ^18^F-FDG. One hour after intravenous injection of 370 MBq/kg ^18^F-FDG W was imaged on a PET/MR hybrid scanner. For the ^11^C-flumazenil PET/ MR brain scan W received a 370 MBq intravenously injected bolus of ^11^C-flumazenil at the start of the 90 minute scan, followed by a 70 minute continuous infusion with an initial activity of 130MBq. High resolution structural T1-weighted volumetric MR images were acquired simultaneously to the PET images, using a 3 Tesla Siemens PET/ MR hybrid system.

All the scans with zolpidem was performed one hour after the consumption of 10mg zolpidem.

Scans were co-registered at a similar section thickness, using anatomical MR data for reproducible positioning. The ^18^F-FDG PET and ^11^C-flumazenil images are shown off and on zolpidem, as are the ^99m^Tc-HMPAO brain SPECT images of 2011 and 2014.
